# Sequential Metastatic Breast Cancer Chemotherapy: Should the Median be the Message?

**DOI:** 10.3389/fpubh.2013.00049

**Published:** 2013-11-11

**Authors:** Su Yon Jung, Margaret Rosenzweig

**Affiliations:** ^1^Department of Epidemiology, Division of Cancer Prevention and Population Sciences, The University of Texas MD Anderson Cancer Center, Houston, TX, USA; ^2^University of Pittsburgh School of Nursing, Pittsburgh, PA, USA

**Keywords:** metastatic breast cancer, chemotherapy, sequential treatment, survival, treatment counseling

## Abstract

**Background:** Counseling and anticipatory guidance of the expected course of treatment for women newly diagnosed with metastatic breast cancer (MBC) are difficult due to multiple factors influencing survival following MBC therapy. In order to better tailor counseling at the onset and through the duration of MBC we used non-clinical trial data to better characterize real life experience of sequential MBC treatment. We examined the following aims: (1) What demographic and tumor characteristics are predictive of survival in MBC? (2) What is the median duration of each sequential chemotherapy regimen and subsequent survival of women following each sequence of chemotherapy regimen in MBC?

**Methods:** Retrospective study included 792 women diagnosed from January 1999 through December 2009 at the University of Pittsburgh Cancer Institute Breast Cancer Program.

**Results:** Median duration of sequential chemotherapy regimen and median survival from completion of sequence of chemotherapy regimens were relatively short with a wide range of treatment duration and survival. Characteristics for poor survival included hormone status, human epidermal growth factor receptor-2 (HER 2/*neu*) status, and increased number and type of metastatic sites. Women who took more than the second sequential chemotherapy regimens had no more than median 3 months of treatment duration and 6 months survival from treatment termination.

**Discussion:** Median clinical response and survival shorten with sequential chemotherapy regimen but with wide ranges. The rare clinical response of the minority should not set the standard for treatment expectations. All cancer clinicians, including oncology nurses, must ensure that patients are receiving tailored counseling regarding their specific risks and benefits for sequential MBC chemotherapy.

## Introduction

It is estimated that 160,000 United States women are living with metastatic breast cancer (MBC) with median survival of 2–3 years (1). National data base analysis (2, 3) as well as local experience (4) indicates that chemotherapy treatment until the last months before death from MBC is common practice due to multiple etiologies. First, the heterogenic nature of breast cancer and the variability of treatment response allows for several chemotherapies to be utilized for MBC treatment in sequential fashion without clear stopping rules. Next, there are believed to be some modest survival advantages (5) and quality of life benefits in women receiving more, rather than less chemotherapy in MBC. Additionally, it is well documented that in advanced cancer, patients prefer chemotherapy with minimal potential benefit, rather than termination of anti-tumor treatment (6, 7).

Lastly the national consensus guidelines for cancer practice encourage the use of sequential chemotherapy to treat MBC (8). The 2013 National Comprehensive Cancer Network recommendation for systemic metastatic chemotherapy is as follows (8): “Failure to achieve a tumor response to three sequential chemotherapy regimens or an Eastern Cooperative Oncology Group performance status of three or greater was believed to be an indication for supportive therapy only. In this context, failure to respond to a chemotherapy regimen means the absence of even a marginal response to the use of a given chemotherapy regimen. Response to a chemotherapy regimen followed by progression of disease is not considered a failure to experience response.” Essentially this recommendation speaks to wide heterogeneity in treatment response in MBC, even among women who have been heavily treated with chemotherapy.

This wide range of treatment response leads to initial counseling for patients with MBC and it can portray MBC as a “chronic” disease similar to hypertension and diabetes, probably not curable but very treatable. This explanation may be true for specific subsets of women with MBC [low volume disease, estrogen receptor (ER) – positive, human epidermal growth factor receptor-2 (HER 2/*neu*) – positive], but leaves many women poorly prepared for the progressive and ultimately life ending experience of MBC. The available clinical evaluation of chemotherapy in MBC is usually direct comparison of one chemotherapy regimen vs. another to determine survival and clinical benefit endpoints (9–16).In a non-clinical trial environment, the patient clinical experience, and survival benefit of sequential palliative chemotherapy for MBC have not been well described. This leaves little information for the cancer clinician on which to base their treatment counseling for MBC patients. In order to provide a data base for evidence based approach to MBC we conducted a retrospective study with a large clinical data collected for 10 years. Women diagnosed with MBC from this academic based practice were examined to answer the following research questions:

1. What are the influences of demographic and tumor characteristics on survival with MBC?
2. What are the median and ranges of durations of sequential chemotherapy regimens in MBC and what are the median and ranges of survival after the termination of each of last sequential chemotherapy regimen?

## Materials and Methods

This study was based on an Institutional Review Board (IRB) approved protocol for reviewing medical record about women diagnosed with MBC at one large urban practice of the University of Pittsburgh Cancer Institute Breast Cancer Program. Women gave informed consent to having their medical chart reviewed for breast cancer studies with a global consent for cancer registry at the onset of their breast cancer care. The clinic center offers comprehensive counseling and cancer treatment services including administration of chemotherapy, supportive cancer therapies, and psychological counseling. Inclusion criteria included women at 18 or older years of age with MBC diagnosed between January 1999 and December 2009. Second opinion visits were not included.

Patients with MBC were identified from daily clinic lists with disease stage. Medical record review confirmed the diagnosis of MBC through clinical, radiological, or pathologic confirmation. There were 38 demographic and historical items captured with entry into the database with 14 entries abstracted monthly according to a chart abstraction and quality assurance protocol. Among those demographic, pathologic, and clinical variables, for the purpose of this study, following variables were selected to use in analysis: age, race, number of metastatic sites, metastatic location, ER and/or progesterone receptor (ER/PR) status, and HER 2 status. Systemic chemotherapy was evaluated for their sequences. The primary tool for abstraction was the clinic note, usually completed monthly with patient visit. Abstraction was completed by registered nurses with clinical experience in breast cancer.

Bivariate analyses were performed to describe descriptive patterns and relationships among key variables. These analyses included Wilcoxon rank sum test to examine bivariate relationships between variables. Log-rank test and Kaplan–Meier’s graph were conducted to evaluate the relationship between categorical variables and survival. Data were collected and entered into an EXCEL data base and exported to SAS 9.2 for analysis. Two-tailed *P*-value <0.05 was considered significant.

### Operational definitions

- Tumor characteristics: ER/PR and HER 2 status were determined from metastatic sites when available. If metastatic tissue was not present then primary breast tissue reports were used for determining ER/PR and HER 2 status.
- Clinical benefit: it is difficult to measure treatment efficacy during sequential palliative chemotherapy. The standard Response Evaluation Criteria In Solid Tumors (RECIST) criteria or indicators of tumor progression, radiographic progression, or increase in tumor marker, become less relevant if the patient remains clinically stable, or conversely, experiences an increase in symptom ontology without significant tumor progression. The term “clinical benefit” matches the approved language from the National Cancer Institute in determining clinical endpoints for advanced cancer (14). Clinical benefit is measured as the time until need for change in chemotherapy or hormonal therapy due to disease progression or intolerance to therapy rather than formal measures of radiographic progression. These determinations were made by a trained registered nurse reading the sequential monthly clinic notes written by the physician, nurse practitioner or physician assistant and making a protocolized determination regarding the reason for treatment change. Clinical benefit was utilized as a surrogate for time of disease progression.
- Sequential chemotherapy category: patient visits were abstracted monthly at the first visit of the month. Systemic treatments including chemo and hormone therapy were coded and then counted according to months of treatment. A chemotherapy regimen (i.e., a set of chemo drugs) was counted as first, second, etc., regardless of other systemic treatments. For example, if one chemotherapy regimen was initiated as a second treatment, following several months of hormonal therapy, this was counted as first chemotherapy and subsequent duration of therapy and survival following completion of therapy was calculated accordingly.

## Results

### Breast cancer characteristics

The database consists of 792 women diagnosed with MBC from January 1999 through December 2009. In this cohort, 50.4% (*n* = 399) of women were <55 years of age. The majority of the sample was non-African American population (93.2%, *n* = 738). More than 70% (*n* = 560) of women were ER/PR positive with 27.8% women with ER/PR negative status. HER 2 positive status was reported in 261 women (33.0%) and 464 women (58.6%) were reported as negative status. More than half of women had metastasis on bone (67.7%) and visceral site (65.8%), while 26 and 30% of women were presented as brain and soft tissue metastasis, respectively. See Table 1.

**Table 1:** **Survival difference according to demographic and tumor characteristics**.

Variable	No.	(%)	Mean (SE) month^a^	Median month^a^	*P*-value*
**Age^b^**
<55 years	399	(50.4)	44.7 (1.98)	33.0	0.0840
≥55 years	393	(49.6)	36.6 (1.43)	31.0	
**Race**
Non-African American	738	(93.2)	42.6 (1.44)	32.0	0.6970
African American	54	(6.8)	38.0 (4.51)	31.0	
**Number of metastatic sites**					
1	192	(24.3)	52.2 (3.01)	46.0	<0.0001
2	229	(28.9)	42.7 (2.81)	29.0	
3+	371	(46.8)	37.5 (1.54)	32.0	
**Metastatic location**
Bone	536	(67.7)	42.2 (1.57)	33.0	0.8731
Brain	205	(25.9)	36.2 (1.92)	29.0	0.0084
Visceral	521	(65.8)	38.2 (1.47)	30.0	<0.0001
Soft tissue	238	(30.1)	41.6 (2.42)	32.0	0.6067
Other	175	(22.1)	44.4 (2.73)	37.0	0.0851
**ER/PR status^c^**
ER/PR positive	560	(70.7)	46.4 (1.69)	38.0	<0.0001
ER/PR negative	220	(27.8)	32.1 (2.13)	22.0	
**HER 2 status^c^**
HER 2 positive	261	(33.0)	44.6 (2.09)	38.0	0.0199
HER 2 negative	464	(58.6)	40.2 (1.80)	30.0	

*SE, Standard error; ER/PR, estrogen receptor and/or progesterone receptor; HER 2, human epidermal growth factor receptor-2*.

*^a^ Survival was defined as interval in month between metastatic breast cancer diagnosis and death or study end point*.

*Relationship between variable and survival was evaluated using Log-rank test.

*^b^ Age variable was classified using median age (=55 years)*.

*^c^ Unknown ER/PR (*n* = 12) or Her 2 (*n* = 67) were excluded from analysis*.

### Survival relationships with demographic and tumor characteristics

**Figure 1:**
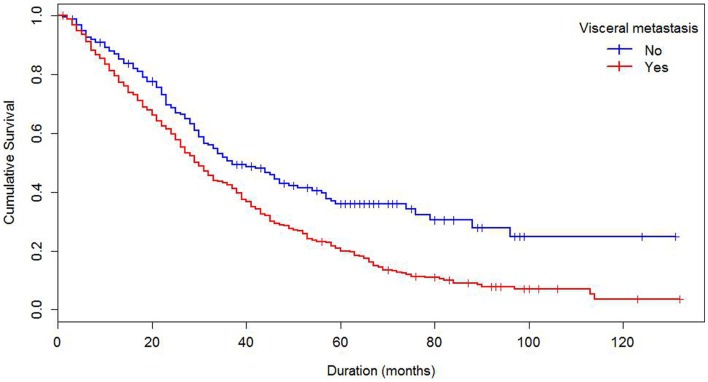
**Kaplan–Meier’s curve of survival by visceral metastasis status**.

**Figure 2:**
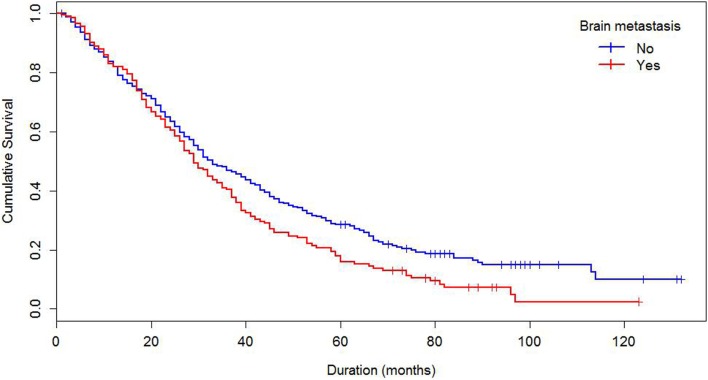
**Kaplan–Meier’s curve of survival by brain metastasis status**.

Median survival of the cohort was 32 months and mean survival 42.5 months (SE 1.4). The 5-year survival rate was 24%. Some demographics and tumor characteristics were associated with shorter survival from the diagnosis of MBC (Table 1). Increasing numbers of metastatic sites, ER/PR negativity, HER 2 negative status were associated with worse survival. Additionally, women with brain and visceral metastasis compared with non-metastasis of the relevant site had poor prognosis (Figures 1 and 2); these relationships were constantly observed when stratified by HER 2 status. Among both women with HER 2 positive and negative status, women without brain and visceral metastasis were likely to have longer survival than women with metastasis of the relative site. In addition, when categorized by ER/PR status, among women with ER/PR positive status, those who did not have brain and visceral metastasis had longer survival than those who had brain and visceral metastasis respectively.

### Sequential chemotherapy regimen

Sequential chemotherapy regimen, independent of other systemic treatments, was analyzed for treatment duration and survival from completion of that set of chemotherapies (Table 2). The number of sequential chemotherapy regimens ranged from 1 chemotherapy regimen treatment through 13 different sequential chemotherapy regimens for MBC. After the first MBC chemotherapy regimen and through subsequent regimens of chemotherapy, median durations of therapy were 2–3 months with ranges from 1 to 81 month durations. Median survivals from discontinuation of sequential chemotherapy regimens were no more than 6 months following the second sequential chemotherapy regimen. Range of survival however was large ranging from 0 to 129 months. Even following sixth line chemotherapy regimen the range of survival extended to 36 months. It thus appears that in MBC, there are decreasing subsets of women who achieve months of clinical benefit and of survival even after receiving multiple sequential regimens of chemotherapy.

**Table 2:** **Distribution of treatment durations of sequential chemotherapy regimen and survival from completion of the sequential treatment**.

Sequence of chemotherapy regimen	No.	(%)	Median month and range of sequential chemotherapy regimen	Median month and range of survival from completion of sequential chemotherapy regimen
1	636	(100)	4.0 (1–64)	13.0 (0–129)
2	443	(69.7)	3.0 (1–33)	7.0 (0–117)
3	304	(47.8)	2.0 (1–24)	5.0 (0–114)
4	201	(31.6)	3.0 (1–81)	5.0 (0–42)
5	129	(20.3)	3.0 (1–17)	4.0 (0–41)
6	85	(13.4)	2.0 (1–16)	3.0 (0–36)
7	51	(8.0)	2.0 (1–11)	3.0 (0–29)
8	27	(4.2)	2.0 (1–12)	2.0 (0–17)
9	17	(2.7)	1.0 (1–5)	2.0 (0–15)
10	7	(1.1)	2.0 (1–6)	6.0 (1–10)
11	4	(0.6)	2.0 (1–6)	3.5 (1–8)
12	2	(0.3)	2.5 (2–3)	3.0 (1–5)
13	2	(0.3)	1.5 (1–2)	1.5 (0–3)

Individual tumor characteristics influence response to chemotherapy. We found that ER/PR and HER status modified the relationships between sequences of chemotherapy regimen and median months of therapy duration and survival from completion of treatment. When data were collapsed into first regimen of chemotherapy, second, and three or greater regimens of chemotherapy, ER/PR status was minimally predictive (Table 3) and HER 2 status was strongly predictive of the median months of chemotherapy regimen duration and median months of survival from completion of chemotherapy regimen treatment (Table 4). Across sequences of chemotherapy regimen, women with HER 2 positive had longer median duration of chemotherapy regimen and improved median survival from the last chemotherapy regimen.

**Table 3:** **Median months of sequential chemotherapy regimen and survival from completion of sequential treatment, stratified by ER/PR status**.

Sequence of chemotherapy regimen	No.	Median month (No.) of sequential chemotherapy regimen					Median month (No.) of survival from completion of sequential chemotherapy regimen				
		ER/PR positive		ER/PR negative		*P*-value*	ER/PR positive		ER/PR negative		*P*-value*
1	626	5.0	(420)	4.0	(206)	0.0283	14.0	(420)	11.0	(206)	0.0891
2	436	4.0	(280)	3.0	(156)	0.0006	9.0	(280)	6.0	(156)	0.0925
3 +	302	6.0	(196)	5.5	(106)	0.4300	7.0	(196)	8.5	(106)	0.9207

*ER/PR, estrogen receptor and/or progesterone receptor*.

*Significant difference of median months of sequential chemotherapy regimen or survival from the sequential treatment between ER/PR positive and negative was evaluated using Wilcoxon rank sum test.

**Table 4:** **Median months of sequential chemotherapy regimen and survival from completion of sequential treatment, stratified by HER 2 status**.

Sequence of chemotherapy regimen	No.	Median month (No.) of sequential chemotherapy regimen					Median month (No.) of survival from completion of sequential chemotherapy regimen				
		HER 2 positive		HER 2 negative		*P*-value*	HER 2 positive		HER 2 negative		*P*-value*
1	598	5.0	(240)	4.0	(358)	0.0018	19.0	(240)	10.5	(358)	<0.0001
2	419	4.0	(173)	3.0	(246)	0.0584	10.0	(173)	6.0	(246)	0.0001
3+	289	9.0	(132)	4.0	(157)	<0.0001	16.0	(132)	4.0	(157)	<0.0001

*HER 2, human epidermal growth factor receptor-2*.

*Significant difference of median months of sequential chemotherapy regimen or survival from the sequential treatment between HER 2 positive and negative was evaluated using Wilcoxon rank sum test.

## Discussion

The results from this study and the current literature illuminate the reasons for the pattern of sequential chemotherapy use among patients with MBC. This heterogeneous response to breast cancer chemotherapy, even in women who have been heavily pre-treated makes generalized predictions about survival particularly difficult, often resulting in overly optimistic physician communication and patients’ poor understanding regarding the reality and impact of their disease status (17). The heterogeneity in response and wide ranges of potential duration of benefit and survival from termination of treatment in a small subset of women has prompted clinicians to base decisions for all women on the survival outliers rather than the median survival, prompting the recommendation of continuation of sequential chemotherapy until close to death.

Looking at these data only in terms of the outliers is problematic. The data indicate that the discontinuation of third metastatic chemotherapy regimen is the point of illness in MBC when the median survival is <6 months. This 6 month prognosis is the classic delineation for initiation of hospice and palliative care (17). Among this cohort however, reliance on median survivals and waiting for this 6 month survival time point for end of life counseling will ignore the needs of almost half of the women who will die prior to that point. Of the cohort who began chemotherapy, only 31.6% (*n* = 201) began fourth line chemotherapy. The end of life needs, such as hospice and palliative care referrals, of the majority cannot be ignored by clinicians who have enthusiasm regarding potential survival benefit for a small minority of women (18–21). Additionally, there are some demographic and tumor characteristics that predict poor response to treatment and overall survival. These variables, including hormone status, HER 2 status, and increased number and type of metastatic sites have been established (22) and were confirmed by this sample. The presence or absence of these variables allows for some tailoring of counseling regarding “what to expect” rather than counseling all women with the same message of “control not cure.” Counseling needs to incorporate the median durations of treatment, and survival with some tailoring for individual characteristic’s, rather than only emphasizing the few women with continued clinical benefit to sequential therapy. Additionally because some women with high risk disease do die quickly from MBC, some counseling regarding the terminal nature of MBC needs to be incorporated at the diagnosis of MBC.

A well-known 1985 essay, “The Median Isn’t the Message” was written by a scientist diagnosed with a rare abdominal mesothelioma and subsequent poor life prognosis (23). He analyzed the probabilities of living longer than the median survival for his specific disease and wrote an essay extolling the virtues of a positive attitude and “raging mightily against the dying of the light (23).” While he enjoyed a 20-year remission of his disease and did fall into the positive tail of the survival curve, it is important to remember that he was an outlier. To counsel all patients to expect to live 20 years when the median survival is much less is not honest and places an undue burden on the patient to “be positive” without strong scientific evidence that attitudes alone can influence the course of disease (24, 25).

These honest conversations that need to occur at the diagnosis of MBC and throughout the course of illness are difficult and require time and skill. The American Society of Clinical Oncology (ASCO) released a statement in January 2011 emphasizing the need for individualized care for all patients with cancer (26). They conclude that conversations regarding “realistic conversations regarding prognosis, the potential benefits of and limitations of disease directed therapy, and the potential role of palliative care,” in conjunction with or as an alternative to disease directed therapy occur late in the cancer therapy and should occur earlier and consistently. While ASCO focuses on the conversations between physician and patient, nurses can be instrumental in ensuring that all women receiving care for MBC are following an individualized plan of care, tailored to their specific risk and care needs. Nurse can assess a women’s understanding of her disease and treatment plan and treatment goals, advocating for patient/physician conversations to occur, encourage patients to discuss questions or concerns with physicians, or in the case of advanced practice nursing, become skilled in these conversations themselves (27).

Ongoing analysis from several MBC cohorts (28, 29) over the course of several decades has shown a progressive increase in MBC survival, attributed in part to more aggressive systemic therapies. Our analysis points out that a small number of women do have wide ranges of survival even with late sequence chemotherapy regimen. The women who have clinical benefit from late sequential therapies of chemotherapy are intriguing and need to be analyzed in a more systematic fashion. This systematic analysis should include demographic and original tumor factors, previous treatment patterns, characteristics of the metastatic disease and possible changes in tumor characteristics throughout MBC therapy. Genomic analysis through micro array and immunohistochemistry technologies may potentially add new information to better understand a favorable response in MBC allowing a more tailored approach to MBC (30).

There are limitations to this analysis. First, the nature of retrospective study and chart abstraction in women who have multiple care providers, even multiple cancer care providers, leaves a large amount of sequential data incomplete, or completely missing. Secondly, the question of clinical benefit must include quality of life, overall patient distress, and symptom scores, none well established through retrospective chart review. Symptom and quality of life assessment must be incorporated in real time clinical practice, using clinically relevant quality of life scales.

## Conclusion

There is not a strong evidence base for sequences of chemotherapy regimen treatment for MBC, making treatment decision and end of life counseling in MBC a challenge for cancer care professionals. It is appropriate to be hopeful about each new therapy, but with counseling that encompasses a “hope for the best but prepare for the worst” (31) content, rather than a “MBC is a chronic illness with unlimited treatment option approach.” Clinicians must also consider that despite some promise of late stage clinical benefit, intensive sequential treatment spanning many months and even years is not without larger emotional and financial consequence for patients and surviving family (31, 32).

The ASCO statement suggests that instead of each new line of chemotherapy becoming the immediate “default” after cancer progression, a new discussion of treatment goals, patient’s understanding of risk and benefit, and a clear explanation of costs including monetary, time, and toxicity be offered. Each sequential chemotherapy regimen then becomes a new decision. ASCO also emphasizes that patients wanting a therapy does not preclude the necessity of having a conversation regarding the likelihood of true benefit from that treatment. These conversations are extremely difficult in MBC due to the occasional outlier in response to a late line of chemotherapy. All patients believe they will be “the one” to respond, rather than believing that it is likely that they will be within the median. Although difficult, it is the clinician’s responsibility to offer hopeful but realistic counseling (33, 34).

These results have implication for (1) greater attention to quality of life endpoints, (2) creation of randomized controlled trials evaluating efficacy of sequential MBC therapies, (3) guidance for initiation of Phase I trials in the MBC treatment course, (4) reassessment of hospice criteria to allow both hospice care and palliative chemotherapy due to the heterogeneity in chemotherapy response and quality of life benefit with palliative chemotherapy, (5) methods to determine financial implication (patient and payer) of sequential MBC palliative care, and (6) incentive to better understand the unique characteristics of the long term MBC survivors for better prognostic and predictive information. This additional information will better inform the clinician charged with patient counseling as metastatic patient counseling treatment commences.

## Conflict of Interest Statement

The authors declare that the research was conducted in the absence of any commercial or financial relationships that could be construed as a potential conflict of interest.
